# Recommendations of the Neuroendocrinology Department of the Brazilian Society of Endocrinology and Metabolism for the diagnosis of Cushing’s disease in Brazil

**DOI:** 10.1590/2359-3997000000174

**Published:** 2016-02-11

**Authors:** Márcio Carlos Machado, Maria Candida Barisson Vilares Fragoso, Ayrton Custódio Moreira, César Luiz Boguszewski, Leonardo Vieira, Luciana A. Naves, Lucio Vilar, Luiz Antônio de Araújo, Mauro A. Czepielewski, Monica R. Gadelha, Nina Rosa Castro Musolino, Paulo Augusto C Miranda, Marcello Delano Bronstein, Antônio Ribeiro-Oliveira

**Affiliations:** 1 Hospital das Clínicas Faculdade de Medicina Universidade de São Paulo São Paulo SP Brasil Unidade de Neuroendocrinologia, Serviço de Endocrinologia e Metabologia, Hospital das Clínicas da Faculdade de Medicina da Universidade de São Paulo (HCFMUSP); Departamento de Endocrinologia, A.C. Camargo Cancer Center, São Paulo, SP, Brasil;; 2 Faculdade de Medicina de Ribeirão Preto Universidade de São Paulo Ribeirão Preto SP Brasil Divisão de Endocrinologia e Metabologia, Faculdade de Medicina de Ribeirão Preto, Universidade de São Paulo (FMRP-USP), Ribeirão Preto, SP, Brasil;; 3 Serviço de Endocrinologia e Metabologia Hospital de Clínicas Universidade Federal do Paraná Curitiba PR Brasil Serviço de Endocrinologia e Metabologia (SEMPR), Hospital de Clínicas, Universidade Federal do Paraná (UFPR), Curitiba, PR, Brasil;; 4 Serviço de Endocrinologia Hospital Universitário Clementino Fraga Filho Universidade Federal do Rio de Janeiro Rio de Janeiro RJ Brasil Serviço de Endocrinologia, Hospital Universitário Clementino Fraga Filho, Universidade Federal do Rio de Janeiro (HUCFF/UFRJ), Rio de Janeiro, RJ, Brasil;; 5 Serviço de Endocrinologia Hospital Universitário de Brasília Universidade de Brasília Brasília DF Brasil Serviço de Endocrinologia, Hospital Universitário de Brasília, Universidade de Brasília (UnB), Brasília, DF, Brasil;; 6 Serviço de Endocrinologia Hospital de Clínicas Universidade Federal de Pernambuco Recife PE Brasil Serviço de Endocrinologia, Hospital de Clínicas, Universidade Federal de Pernambuco (UFPE), Recife, PE, Brasil;; 7 Endoville Joinville SC Brasil Endoville, Joinville, SC, Brasil;; 8 Hospital de Clínicas de Porto Alegre Faculdade de Medicina Universidade Federal do Rio Grande do Sul Porto Alegre RS Brasil Serviço de Endocrinologia, Hospital de Clínicas de Porto Alegre (HCPA), Faculdade de Medicina da Universidade Federal do Rio Grande do Sul (UFRGS), Porto Alegre, RS, Brasil;; 9 Hospital das Clínicas Faculdade de Medicina Universidade de São Paulo São Paulo SP Brasil Unidade de Neuroendocrinologia, Divisão de Neurocirurgia Funcional, Hospital das Clínicas da Faculdade de Medicina da Universidade de São Paulo (HCFMUSP), São Paulo, SP;Brasil; 10 Serviço de Endocrinologia Santa Casa de Belo Horizonte Belo Horizonte MG Brasil Serviço de Endocrinologia, Santa Casa de Belo Horizonte, Belo Horizonte, MG, Brasil;; 11 Universidade Federal de Minas Gerais Serviço de Endocrinologia Hospital de Clínicas Belo Horizonte MG Brasil Serviço de Endocrinologia, Hospital de Clínicas, Universidade Federal de Minas Gerais (UFMG), Belo Horizonte, MG, Brasil

**Keywords:** Cushing’s disease, Cushing’s syndrome, diagnosis

## Abstract

Although it is a rare condition, the accurate diagnosis and treatment of Cushing’s disease is important due to its higher morbidity and mortality compared to the general population, which is attributed to cardiovascular diseases, diabetes mellitus and infections. Screening for hypercortisolism is recommended for patients who present multiple and progressive clinical signs and symptoms, especially those who are considered to be more specific to Cushing’s syndrome, abnormal findings relative to age (*e.g.*, spinal osteoporosis and high blood pressure in young patients), weight gain associated with reduced growth rate in the pediatric population and for those with adrenal incidentalomas. Routine screening is not recommended for other groups of patients, such as those with obesity or diabetes mellitus. Magnetic resonance imaging (MRI) of the pituitary, the corticotropin-releasing hormone (CRH) test and the high-dose dexamethasone suppression test are the main tests for the differential diagnosis of ACTH-dependent Cushing’s syndrome. Bilateral and simultaneous petrosal sinus sampling is the gold standard method and is performed when the triad of initial tests is inconclusive, doubtful or conflicting. The aim of this article is to provide information on the early detection and establishment of a proper diagnosis of Cushing’s disease, recommending follow-up of these patients at experienced referral centers. Arch Endocrinol Metab. 2016;60(3):267-86

## INTRODUCTION

Endogenous Cushing’s syndrome can be defined as a condition resulting from prolonged and inappropriate exposure to excessive amounts of cortisol, partial loss of the normal counter-regulation of the hypothalamic-pituitary-adrenal axis and loss of circadian rhythm in cortisol secretion (1).

Cushing’s disease (CD) is a rare clinical condition that is due to ACTH-producing pituitary adenoma, and it is the most common etiology of endogenous Cushing’s syndrome after 6 yrs of age (~70%) (2).

CD has an estimated incidence of 2-3 cases per 1.000.000 inhabitants/year and a prevalence of approximately 40 cases per 1.000.000 inhabitants (3), occurring mainly in women (3-8: 1) (3,4). It can affect individuals at any age, but it is most prevalent in the second and third decades of life and is mostly (~80-90%) caused by pituitary tumors with a diameter less than 10 mm (microadenomas) (5-7).

ACTH-secreting pituitary adenomas are sporadic tumors in the vast majority of cases, but they can rarely be part of genetic conditions such as multiple endocrine neoplasia type 1 (NEM1), multiple endocrine neoplasia type 4 (NEM4) and familial isolated pituitary adenoma (FIPA) (8). Other less common etiologies of endogenous Cushing’s syndrome include ectopic ACTH secretion (EAS ~10%), adrenal adenomas and carcinomas and, more rarely, adrenal primary macronodular adrenal hyperplasia (PMAH) or primary pigmented nodular adrenal disease (PPNAD) (Table 1). In children under 5 years of age, adrenal causes (ACTH-independent: adrenal adenoma and carcinoma) are the most common, whereas bilateral adrenal hyperplasia due to McCune-Albright syndrome is the most common cause in the first year of life. CD is the most prevalent etiology (75%) in patients older than 7 years (2).

**Table 1:** Etiologies of endogenous Cushing’s syndrome

Cushing’s syndrome etiology*	Prevalence**
**ACTH-dependent Cushing’s syndrome**	**80%**
Cushing’s disease	70%
Ectopic ACTH syndrome	10%
Ectopic CRH secretion	< 1%
Carcinoma corticotropic	Rare
Ectopic CRH/ACTH secretion	Rare
**ACTH-independent Cushing’s syndrome**	**20%**
Adrenal adenoma	10%
Adrenal carcinoma	5%
Primary macronodular adrenal hyperplasia (PMAH)	< 2%
Primary pigmented nodular adrenocortical disease (PPNAD)	< 2%
McCune-Albright syndrome	Rare
Ectopic cortisol secretion	Rare
Cortisol hypersensitivity	Rare

* References: 4,49,117,182; ** Prevalence in adult patients.

Patients with Cushing’s syndrome have a higher mortality than the general population (9-21), mainly due to the development of cardiovascular disease (ischemic heart disease and cerebrovascular disease), diabetes mellitus (secondary to hypercortisolism) and infections (due to the state of immunosuppression).

A meta-analysis showed a standardized mortality ratio (SMR) of 2.22 (1.45-3.41; confidence interval 95%) (15), which varied between studies from 0.98 (0.44-2.18) (10) to 4.80 (2.79 to 8.27) (15). However, when analyzing the mortality rate in relation to the remission of Cushing’s syndrome, there was a higher mortality rate in patients with persistent disease compared to those in clinical remission: 5.50 (2.69-11.26) *vs*. 1.20 (0.45-3.18), respectively (15).

Another factor that contributes to the onset and progression of the associated comorbidities is the difficulty in identifying patients with a subtler clinical picture, which delays the diagnosis of Cushing’s syndrome and its etiology. Some studies have shown that this time can vary from 2-5 years from the initial consultation to the end of investigation (9,22-25), and the longer exposure to hypercortisolism has been proved to be an independent factor associated with higher mortality in patients with Cushing’s syndrome (17).

Thus, to improve the prognosis of patients with Cushing’s syndrome and to enhance the reversibility of comorbidities, an early recognition of the disease and the reversal of hypercortisolism to eucortisolism are essential. Eventually, very severe hypercortisolism may present as a medical emergency with very high mortality if not promptly treated. The diagnosis of these patients must be made expeditiously, and treatment must be started immediately (1).

The aim of this article is to provide information on the early detection and establishment of a proper diagnosis of CD. We recommend monitoring these patients in centers with experienced multidisciplinary teams (endocrinologists, neurosurgeons, radiologists, radio-interventionists) to establish the diagnosis, to indicate the best treatment and to conduct follow-ups of these patients.

### DIAGNOSIS OF ENDOGENOUS CUSHING’S SYNDROME

#### Who should we investigate for Cushing’s syndrome?

The classic clinical features of Cushing’s syndrome consist of weight gain causing overweight or obesity with an abdominal and truncal distribution, fatigue, menstrual abnormalities such as oligomenorrhea or amenorrhea, reduced libido and/or erectile dysfunction, decreased growth rate associated with weight gain in children, and psychiatric disturbances including depression, decreased concentration and memory, irritability and insomnia.

Physical examinations can show a wide variability of features related to Cushing’s syndrome, ranging from subtle to overt features. In patients who present with overt Cushing’s syndrome, we can observe plethoric round face (“moon face”), dorsal hump (“buffalo hump”), filled supraclavicular fossa, skin atrophy, acne, hirsutism, hair loss, and peripheral edema. In children, a short stature, abnormal virilization, pubertal delay or pseudo precocious puberty may be observed. Moreover, there are common comorbidities in clinical practice, such as hypertension, diabetes mellitus, nephrolithiasis, osteopenia or osteoporosis, hypokalemia, and unusual fungal infections. However, all of these signs, symptoms and morbidities are non-specific, as they may be present in metabolic syndrome, severe diabetes mellitus, polycystic ovary syndrome, grade III obesity, depression and chronic alcoholism (26). Thus, it is important to look for more specific signs of Cushing’s syndrome, such as facial plethora, proximal muscle weakness, large (> 1 cm) and reddish/violet skin striae and spontaneous ecchymosis in patients presenting weight gain (27,28) (Table 2).

**Table 2:** Prevalence of symptoms, signs and morbidities of Cushing’s syndrome

Symptoms, signs and morbidities*	Prevalence %
Weight gain or obesity/abdominal obesity	95
Facial plethora	90
Facial fullness	90
Decreased libido/erectile dysfunction	90
Thin skin	85
Menstrual abnormalities/amenorrhea	80
Decreasing growth velocity**	70-80
Arterial hypertension	75
Hirsutism	75
Depression/emotional lability	70
Dyslipidemia	70
Striae (especially if red or purple and more than 10 mm wide)	70-90
Dorsocervical fat pad/supraclavicular fullness	50-70
Easy bruising	65
Impaired glucose-tolerance/diabetes mellitus	60
Proximal myopathy/weakness	60
Osteopenia or osteoporosis/fracture	50
Kidney stones	50
Exophthalmos	45
Polyuria	30
Headache	20-50
Back pain	20-50
Peripheral edema	20-50
Unusual infections/fungal infections	20-50
Hypokalemia	10-70
Acne	< 20
Alopecia/female balding	< 20
Hyperpigmentation	10

* References: 1,27,49,119,183,184; ** Prevalence in pediatric patients (184).

In contrast, patients suffering from Cushing’s syndrome due to EAS, particularly those with malignancies such as small cell lung carcinoma, may have an atypical clinical presentation, with a predomination of consumptive state (35%), higher frequency of hypokalemia (> 70%), hyperpigmentation, osteopenia/osteoporosis and metabolic disorders, such as glucose intolerance (29).

Thus, it is recommended to investigate Cushing’s syndrome in patients who have multiple and progressive signs and symptoms, especially those considered more specific, such as the presence of spinal osteoporosis and hypertension, weight gain in children coupled with a reduction in growth, and the finding of adrenal incidentalomas. Routine investigation is not otherwise recommended for other groups of patients, such as those with isolated obesity or hirsutism (27,30,31) (Table 3).

**Table 3:** Recommendations for Cushing’s syndrome screening

Cushing’s syndrome screening*
Presence of multiple and progressive features, especially those more specific to Cushing’s**
Unusual features for age (vertebral osteoporosis, arterial hypertension)
Pediatric patients with decreasing growth velocity/short stature and weight gain
Adrenal incidentaloma
We do not recommend widespread screening for Cushing’s syndrome in other clinical situations (e.g., obesity, diabetes mellitus, hirsutism) without the presence of more specific features of hypercortisolism

* References: 27,30,31; ** Easy bruising, facial plethora, proximal myopathy/weakness, striae (especially if red or purple and more than 1 cm wide).

However, several studies have been devoted to investigating hypercortisolism in groups of patients considered at risk or in whom the prevalence of Cushing’s syndrome may be greater than expected. The at-risk conditions often highlighted are secondary hypertension, in which the prevalence of Cushing’s syndrome has been reported to be approximately 0.5-1% of cases, adrenal incidentalomas (6-9%) and unexplained osteoporosis with vertebral fracture (11%) (32,33).

However, the most studied risk condition is diabetes mellitus, which was found to have a prevalence of 0.8% (0-3.3%) in a group of 2,381 diabetic patients from 12 studies (34-45). Striking Cushing’s features have not been described in diabetic patients with an HbA1c between 8.4-12.2% associated with hypertension and overweight/obesity (BMI between 25.4-34.5 kg/m^2^). Additionally, other factors such as the lack of knowledge of the natural history of this “occult” Cushing’s syndrome and questions regarding the best therapeutic strategy (identification and treatment of Cushing’s syndrome *vs*. treatment of diabetes mellitus) preclude a recommendation for routine screening of Cushing’s syndrome in patients with diabetes mellitus (46).

Finally, although there is no indication of the need to screen for Cushing’s syndrome in either overweight or obesity patients without other suggestive features (27-30), one study showed a prevalence of 0.8% of Cushing’s syndrome in 783 obese preoperative patients undergoing bariatric surgery (47). Interestingly, obese patients usually show a normal circadian rhythm of salivary cortisol (48).

#### First-line tests

After clinical suspicion and thorough exhaustive exclusion of exogenous sources of glucocorticoids of any kind, such as oral, injected, topical or inhaled steroids, the diagnosis of Cushing’s syndrome has two sequential steps. The first step consists of tests to confirm hypercortisolism associated with loss of normal circadian rhythm of cortisol secretion and the relative autonomy of cortisol production, independent of the etiology of Cushing’s syndrome. It is noteworthy that at least two distinct methods must be abnormal to diagnose Cushing’s syndrome. Altered findings from only one method may be present in cases of pseudo-Cushing’s.

In a subsequent step, which is usually performed at referral centers, the establishment of the differential diagnosis of ACTH-dependent or independent Cushing’s syndrome should be conducted (49).

The diagnosis of Cushing’s syndrome is very much dependent on cortisol tests, which vary substantially depending on the utilized assay. Therefore, clinicians involved in this diagnosis should be aware of their institutional assays before adhering to the strict cut-offs suggested by the literature.

#### Low-dose dexamethasone suppression test

The low-dose dexamethasone suppression test (LDT) constitutes one of the main methods used for screening, and it evaluates for a lack of negative feedback of cortisol on the hypothalamic-pituitary-adrenal axis. This test should be performed after an overnight oral intake (between 11:00 pm and 12:00 am) of 1 mg dexamethasone, and blood collection for measurement of serum cortisol should occur in the subsequent morning between 8:00 am and 9:00 am. Cortisol values above 1.8 μg/dL (50 nmol/L) are considered abnormal, with a sensitivity higher than 95% and an 80% specificity (27) (Table 4).

**Table 4:** Laboratory methods for Cushing’s syndrome diagnosis

Method	Reference value	Sensitivity %	Specificity %
First-line methods			
Low-dose dexamethasone suppression test - 1 mg overnight (serum cortisol) (27)	> 1.8 µg/dL	> 95	80
Longer low-dose dexamethasone suppression test - 2 mg/day for 48 h – 0.5 mg 6/6 h (serum cortisol) (68)*	> 1.8 µg/dL	92-100	92-100
Late night salivary cortisol (µg/dL or ng/dL or mmol/L) (56)	> 2X ULNR	88-100	82-100
Urinary free cortisol 24 h (µg/24 h) (68)	> 3-4X ULNR	90-98	45-95
Other methods (second-line)			
Late-night serum cortisol (patient awake) (78)	> 7.5 µg/dL	96	100
Ovine CRH after longer low-dose dexamethasone suppression test (serum cortisol) (81-84)	> 1.4 µg/dL (15’)	< 100	< 100
Human CRH test (plasma ACTH, pg/mL; serum cortisol, µg/dL) (86)	Peak > 54 pg/mL and > 12 µg/dL (baseline)	91.3	98.2
Desmopressin test (plasma ACTH, pg/mL; serum cortisol, µg/dL) (90)	∆ > 18 pg/mL and > 12 µg/dL (baseline)	86.6-100	92.8

∆: delta: peak less baseline value; ULNR: upper limit of normal range; * Meta-analysis showed a similar or lower accuracy than that of the low-dose dexamethasone suppression test (1 mg overnight) (54); serum cortisol: μg/dL; to nmol/L, multiply by 27.59; ACTH: pg/mL; to pmol/L, multiply by 0.2202; urinary cortisol: μg/24 h; to nmol/24 h, multiply by 2.759.

Previously, the cutoff value was higher than 5 μg/dL (140 nmol/L), which is still currently used by some authors in some specific clinical situations (*e.g*., adrenal incidentalomas). However, although this criterion increases the specificity of the method (50), the present approach is more adequate and sensitive because it is known that 18% of patients with Cushing’s syndrome have suppressed values below 5 μg/dL, and up to 8% of patients with Cushing’s syndrome have suppressed serum cortisol below 1.8 μg/dL with 1 mg dexamethasone (51).

False positives can occur in states of hyperactivation of the hypothalamic-pituitary adrenal axis (pseudo-Cushing’s state, such as depression and alcoholism) and in conditions increasing cortisol-binding globulin (CBG) such as estrogen treatment, which accounts for up to 50% of the false positives and requires interruption for at least 6 weeks before the test. Other false positive results may occur due to pregnancy, mitotane use, malabsorption of medication or conditions that increase the metabolism of dexamethasone due to activation of the enzyme CYP3A4 (phenytoin, phenobarbital, rifampin, carbamazepine, pioglitazone, topiramate etc.) (Table 5). False negatives can occur in “mild” Cushing’s syndrome and with the use of drugs that reduce the action of the enzyme CYP3A4 (fluoxetine, cimetidine, itraconazole, ritonavir, diltiazem, and amiodarone, among others). Moreover, even drugs that are not traditionally known for changes in the metabolism of dexamethasone may do so as a result of an interaction with other drugs. A complete list of medications can be found on the following site: http://medicine.iupui.edu/flockhart/table.htm.

**Table 5:** Drugs that may interfere with the evaluation of dexamethasone suppression tests

Drugs that accelerate dexamethasone metabolism – induction of CYP 3A4 – potential false positive LDT
Carbamazepine
Ethosuximide
Phenytoin
Phenobarbital
Pioglitazone
Primidone
Rifampicin
Rifapentine

**Drugs that impair dexamethasone metabolism – inhibition of CYP 3A4 – potential false negative LDT**

Amiodarone
Aprepitant/fosaprepitant
Cimetidine
Ciprofloxacin/norfloxacin
Diltiazem
Fluoxetine
Itraconazole/fluconazole
Ritonavir/indinavir/nelfinavir

LDT: low-dose dexamethasone suppression test. Reference: 27. More complete data available on the following site: http://medicine.iupui.edu/flockhart/table.htm.

Likewise, a recent study assessing the influence of the use of several concomitant medications on oCRH stimulation after longer low-dose dexamethasone suppression tests confirmed a low accuracy of the method in patients receiving those medications (52).

As a result, serum dexamethasone concentrations (1 mg overnight: > 140-220 ng/mL) (27,30) that are measured at the same time as serum cortisol can rule out possible confounding factors, increasing the reliability of the method. However, the measurement of dexamethasone is rarely available in our country.

There is no evidence that the use of higher doses of overnight dexamethasone (1.5 or 2 mg) increases the accuracy of the method. Furthermore, the same procedure used in the adult population has been applied to pediatric populations, with the only change being a dose adjustment (15-20 µg/kg) for patients weighing less than 40 kg (2,53).

Alternatively, some authors prefer a low-dose dexamethasone test using fractionated doses rather than 1 mg overnight to increase the specificity of the method in states of hyperactivation of the hypothalamic-pituitary-adrenal axis, such as depression, alcoholism or even uncontrolled diabetes mellitus. In such cases, 0.5 mg of dexamethasone is administered every 6 hours for two days (8 doses), most commonly beginning with the first dose on the first day at 9:00 am and the last dose occurring at 3:00 am on the last day (6 hours before blood collection at 9:00 am) or with the first dose at lunch (12:00 pm) on the first day and the last dose at 6:00 am on the last day (two hours before cortisol collection). Conceived by Liddle in the 1960s and using cortisol metabolite in the urine (17OHCS), the same criterion of a serum cortisol response > 1.8 µg/dL is currently adopted. The non-fractionated 1 mg test is preferable to the 48-h fractionated one because the latter is labor intensive and more error-prone and has shown a diagnostic accuracy slightly lower than that of the overnight test in an important meta-analysis (54).

Thus, rather than choosing the form of dexamethasone administration, it is important to emphasize that at this stage of diagnosis, a greater sensitivity of the methods should be prioritized and, especially in cases of mild Cushing’s, the complementarity and agreement of different methods will confirm the diagnosis, as just one abnormal result does not confirm the diagnosis of the syndrome.

#### Late-night salivary cortisol

Late-night salivary cortisol is an important method in the diagnostic evaluation of Cushing’s syndrome. It should be ordered whenever available, especially in centers with an established methodology and cutoff values that have been studied in different populations (normal, obese/pseudo-Cushing’s and Cushing’s syndrome), with a sensitivity of 88-100% and specificity of 82-100% (55,56) for adults, and of 95.2 and 100%, respectively, for children (53).

An above normal value on this test reflects the lack of a normal circadian rhythm of cortisol secretion, which is considered one of the first events to occur in Cushing’s syndrome. Therefore, some authors have advocated using this test as a first method of screening (33,56-58).

In a study of 11 cases of mild Cushing’s that had a difficult diagnosis, the measurement of late-night salivary cortisol proved to be persistently altered in most patients, while most of the urinary cortisol samples remained within the normal range (59). Another recent study found a higher diagnostic accuracy of nocturnal salivary cortisol when compared to urinary cortisol in 52 patients with Cushing’s syndrome (60).

Salivary cortisol is an analysis method that assesses free cortisol, in dynamic equilibrium with serum cortisol, reflecting a percentage of total serum cortisol (65%), and it is not influenced by saliva flow (61). There are several advantages of this method, such as the non-invasiveness of its collection and the sample stability at room temperature (either one week at room temperature or a few weeks if refrigerated).

Salivary cortisol can be collected in two ways: passive collection (best for small children) or through a commercial collector (Salivette^®^) between 11:00 pm and 12:00 am. It is recommended to collect at least two samples on consecutive or alternate days (62). Salivary cortisol concentrations are commonly determined by radioimmunoassay, ELISA, and automated electrochemiluminescence (62,63); more recently, liquid chromatography/mass spectrometry has also been utilized (30,64-66).

It must be emphasized that a significant conversion of cortisol to cortisone occurs in the salivary glands by the action of 11β-hydroxysteroid dehydrogenase II enzyme (11β-HSD2), leading to a higher concentration of cortisone than cortisol in the saliva. This fact could explain the finding of the same (92%) or even lower (74.5%) sensitivity with equal specificity (90-92%) of salivary cortisol by liquid chromatography/mass spectrometry (LC-MS/MS) in diagnosing Cushing’s syndrome (64-65) compared with the sensitivity and specificity of conventional immunoassays using anticortisol antibodies, showing cross-reactivity with cortisone (55,67). The use of LC-MS/MS can be particularly useful in cases of saliva contamination with synthetic steroids (67).

Because different studies have used different cutoff values with different methods, there is no cutoff value for salivary cortisol that can be widely recommended, unlike the interpretation of serum cortisol after low-dose dexamethasone. Late-night salivary cortisol values more than twice the upper limit have been found to increase the specificity of the method in diagnosing hypercortisolism (67). A review found a mean late-night salivary cortisol level of 250 ± 104 ng/dL (130-415 ng/dL) in patients with Cushing’s syndrome (68).

As with any method, false negatives and most especially false positives can occur in individuals with an altered sleep-wake cycle, psychiatric disorders, uncontrolled diabetes mellitus, and oral/gum disease (blood contamination) and in the elderly (27,37,55); additionally, extremely high results can be due to contamination with corticosteroids in skin creams (57). In addition, it is recommended not to smoke 24 h before collection due to glycyrrhizic acid (derived from licorice), which inhibits the 11β-HSD2 salivary enzyme.

Finally, there have been studies on using morning salivary cortisol levels after an overnight ingestion of 1 mg dexamethasone rather than a traditional serum cortisol test (37,69,70). Given the ease, convenience and noninvasive method of its collection, this may be an alternative to initial screening as it easily comprises two screening methods (late-night salivary cortisol from the previous night and subsequent salivary cortisol after dexamethasone suppression). However, to implement a method of salivary cortisol after dexamethasone suppression, the cross-reactivity of dexamethasone and salivary cortisol should be first ruled out for the chosen assay.

#### 24-h urinary cortisol

The measurement of free cortisol in 24-h urine samples (24-h UFC) as well as serum cortisol suppression tests after low-dose dexamethasone are the traditional diagnostic methods used for the diagnosis and monitoring of patients with Cushing’s syndrome. The 24-h UFC measurement reflects the integrated daily production of cortisol, which is almost always elevated in hypercortisolism.

At least two samples of 24-h UFC, consecutive or alternate, must be requested to exclude false negatives due to variations in cortisol secretion, and 24-h UFC should always be evaluated together with a 24-h creatinine ratio to confirm the adequacy of the sample.

One study found at least one normal sample of 24-h UFC out of four samples in 11% of patients with hypercortisolism (71), and other recent studies have shown a variation of 31-52% in urinary cortisol concentrations in 3-4 samples obtained from the same patients at diagnosis (60,72). Each sample must be collected for 24 h, discarding the first urine sample and including the first urine of the morning the subsequent day. The sample must be kept refrigerated until it is delivered to the laboratory.

False positives can occur in states of pseudo-Cushing’s, such as depression, alcoholism, obesity, pregnancy, and polyuria (example: patients with diabetes insipidus), by the interference of certain drugs (carbamazepine, fenofibrate, digoxin, some synthetic corticosteroids) and other substances that inhibit the 11β-HSD2 enzyme (licorice, carbenoxolone). However, in these cases, the 24-h UFC concentrations are usually less than 1.5-2 times above the upper limit of the method. False negatives may occur in patients with renal insufficiency (creatinine clearance less than 60 mL/minute), mainly due to an inadequate collection of urine (27).

In addition, slightly elevated or normal samples also occur in mild Cushing’s syndrome, adrenal incidentalomas, cyclic Cushing’s syndrome and ACTH-secreting macroadenomas. Currently, considering the data from the three first-line methods, urinary cortisol has been less valued than the others as a screening test for the diagnosis of Cushing’s syndrome (67,73). Likely, a slight increase in cortisol production in the circadian nadir may not be detected as an increase in 24-h UFC. Nevertheless, it remains an important method when used in combination with other diagnostic methods, although it is especially valued and more specific when the observed result is 3-4 times greater than the upper limit of the method.

It is noteworthy to mention that the same reference values for adults can be used in children over 45 kg, with a sensitivity of approximately 89% (74,75).

In general, 24-h UFC does not experience interference from conditions that increase corticosteroid-binding globulin (CBG). There are several methods used to measure 24-h UFC, in particular immunoassays with upper limits of approximately 90-120 µg/24 h. Recently, and similarly to other steroids, more specific methods have been used, such as high performance liquid chromatography (HPLC) and mass spectrometry, with upper limits typically ranging from approximately 40 to 60 µg/24 h. One study showed differences in the value of 24-h UFC in relation to gender, with slightly higher values in women (76), while other studies have found the opposite (77).

#### Additional tests or second-line tests

Second-line tests are indicated when diagnostic uncertainty persists after the completion of first-line tests. This is particularly true for cases of mild Cushing’s syndrome in which the complementarity and correlation of different methods is necessary for a confirmed diagnosis of hypercortisolism (54).

#### Late-night serum cortisol

Although it is an older method and has the same rationale as the nocturnal salivary cortisol test, late-night serum cortisol is considered a second-line examination because it requires the hospitalization of the patient for sample collection at least 48 hours after admission, between 11:00 pm and 12:00 am.

Several studies have analyzed this method, but with different control groups, different collection methods (patient asleep or awake) and with different cutoff values. The most commonly used cutoff value in the literature originates from a 1998 study indicating that a cortisol value higher than 7.5 µg/dL is suggestive of Cushing’s syndrome (patient at rest, but awake) with 96% sensitivity and 100% specificity (78).

A previous study performed collection in sleeping patients (with previously installed venous access) and found a cortisol value higher than 1.8 µg/dL as suggestive of Cushing’s syndrome with 100% sensitivity (79). However, because collection is not always easy in a sleeping patient and the cutoff was very low, other studies have not replicated these data with enough specificity (80). Due to the increased availability and the advantages of late-night salivary cortisol over late-night serum cortisol, the latter is currently seldom used.

Alternatively, and with the same goal, serum cortisol at 4:00 pm has also been analyzed. However, there is a substantial overlap in cortisol values between patients with or without Cushing’s syndrome, preventing an accurate interpretation of the serum cortisol collected at this time point.

#### Ovine CRH stimulation after longer low-dose dexamethasone suppression test

Also known as the Yanovski test, the ovine CRH test was published in 1993 (81). It is still considered by some authors as the best method for the differential diagnosis between Cushing’s syndrome and states of pseudo-Cushing’s. It is performed with 0.5 mg dexamethasone suppression for two days (8 doses) with the last dose at 6:00 am. Then, after two hours, there is an IV infusion of 1 µg/kg or 100 µg of ovine CRH (oCRH). A serum cortisol value higher than 1.4 µg/dL (absolute value) after 15 minutes is considered positive and suggestive of Cushing’s syndrome. In the original publication, this method showed 100% accuracy, an exceptional result, although this has not been reproduced by others (52,82-84). Furthermore, due to the high cost and unavailability of ovine CRH, its use is currently limited. Therefore, we do not recommend this poorly characterized, expensive and complex test.

#### Human CRH test

Traditionally used for the differential diagnosis of ACTH-dependent Cushing’s syndrome (85), the human CRH (hCRH) test was studied in 2009 to differentiate Cushing’s syndrome from states of pseudo-Cushing’s (86). Using statistical analysis to obtain maximum accuracy, the best criterion found has been a serum cortisol value higher than 12 µg/dL at baseline (absolute value, mean time -15 and 0 min) and peak ACTH higher than 54 pg/mL (absolute value) after a 100 µg IV infusion of hCRH, with a sensitivity of 91.3% and specificity of 98.2% for CD, which was better than both first-line methods and nocturnal serum cortisol. However, as with oCRH, due to the high cost and unavailability of the product in Brazil, its use is still currently limited.

#### Desmopressin test

Desmopressin has been used for the differential diagnosis of ACTH-dependent Cushing’s syndrome since 1993 (87). Moreover, it has also been studied with the aim of differentiating Cushing’s syndrome from states of pseudo-Cushing’s (88).

However, as of 2000, the desmopressin test (10 µg IV) showed no significant accuracy in ruling out pseudo-Cushing’s states due to a frequent response (> 50%) in normal subjects when using percentage increases in both ACTH and cortisol as the response criteria (89). In 2007, a study showed equal accuracy between the desmopressin and Yanovski test, but it used the criteria of an ACTH ∆ > 27 pg/mL after desmopressin (84 *vs*. 85%, respectively) (50).

Similar to the hCRH test previously reported (86), the same group published a new reassessment of the desmopressin test after an intensive statistical analysis in 2010, finding new response criteria: serum cortisol higher than 12 µg/dL at baseline (mean between -15 and 0 min) and an ACTH increase of > 18 pg/mL (peak until 30 min minus the baseline value). Using this criteria, a sensitivity of 86.6-100% and a specificity of 92.8% was found, higher than that of the previous criteria of ∆ > 27 pg/mL and again better than the use of increase-percentage values in both ACTH and cortisol. Importantly, this study provided a differential diagnosis of mild Cushing’s syndrome *vs*. pseudo-Cushing’s, providing an advantage when compared to first-line methods and the late-night serum cortisol test (90).

In 2011, one study compared the hCRH with the desmopressin test using the new criteria and verified the identical and excellent performance of both tests in the differential diagnosis of ACTH-dependent Cushing’s syndrome and states of pseudo-Cushing’s, with a 96.6% sensitivity and 100% specificity for both tests (91), results even better than both first-line methods and nocturnal serum cortisol.

Finally, a recent study in patients with CD (*n *= 68) and patients with pseudo-Cushing’s (*n *= 56) demonstrated by ROC curves the use of a peak ACTH value of 71.8 pg/mL after desmopressin, showing a 94.6% specificity, 90.8% sensitivity, 89.8% negative predictive value (NPV) and 95.3% positive predictive value (PPV) for the diagnosis of CD. In the same study, an increase of ACTH equal to or greater than 37 pg/mL after desmopressin showed 88% sensitivity, 96.4% specificity, 87% NPV and 95.3% PPV also for the diagnosis of CD (92).

Therefore, the desmopressin test seems to be a good method for the differential diagnosis of ACTH-dependent Cushing’s syndrome and states of pseudo-Cushing’s. Due to the greater availability and lower cost of desmopressin, it should be further studied.

#### Special situations

***Pregnancy***

The diagnosis of Cushing’s syndrome, though rarely concomitant with pregnancy (93,94), presents unique difficulties. Adrenal adenoma is the most common etiology, accounting for 46% of Cushing’s syndrome cases in pregnancy (93,94). It is also occasionally a cause of pseudo-Cushing’s (95,96).

False positive results commonly occur with low-dose dexamethasone suppression tests in pregnancy due to a secondary CBG increase related to estrogen levels during pregnancy as well as the attenuation of axis suppression in pregnancy (93,94). Regarding 24-h UFC, although not influenced by CBG increase, an important physiological increase in urinary cortisol concentrations in the second and third trimesters has been shown, which can reach up to three times the upper limit of the method (97). Thus, only concentrations higher than 3-4 times the upper limit of the method can be useful for diagnosis.

Typically, the circadian rhythm of cortisol secretion is maintained during gestation and should theoretically be explored for diagnosis. However, there are no specific studies that define cutoff values for late-night serum cortisol during pregnancy, and there are only two studies that assess late-night salivary cortisol with a small group of pregnant women (98,99). Therefore, studies are needed to validate this method in pregnant women. There are also no specific data on the use of CRH or desmopressin in this population.

***Epilepsy***

In the subgroup of individuals with epilepsy, the best methods are late-night salivary/serum cortisol and 24-h UFC. Cortisol suppression tests with low-dose dexamethasone should be avoided due to an interference with dexamethasone metabolism by antiepileptic medications such as phenytoin, phenobarbital and carbamazepine, among others. In addition, carbamazepine metabolites can cause false positives results in 24-h UFC when analyzed by HPLC. One way to improve this accuracy is a concomitant dosage of serum dexamethasone, which should be higher than 140-220 ng/dL after 1 mg overnight intake (27,30), although its availability is limited.

***Renal insufficiency***

Traditionally, 24-h UFC should not be used for patients with renal insufficiency due to their decreased renal excretion with a reduced creatinine clearance of less than 60 ml/min. In this subgroup of patients, late-night salivary cortisol or late-night serum cortisol should be used instead (100). A low-dose dexamethasone suppression test can be used with the same cutoff value of 1.8 µg/dL. However, with progressed reduction in creatinine clearance, there is a decreased excretion of dexamethasone, which may then lead to false negative results.

***Cyclical Cushing’s syndrome***

The assessment of cyclical Cushing’s syndrome is a major diagnostic and therapeutic challenge. This challenge is in part due to its low frequency, at approximately 15% of cases (101), but is also due to a great variability in the duration and interval between cycles, which can vary from days to years (101,102). Importantly, diagnostic and etiological research should only be carried out in the presence of active hypercortisolism because tests are negative during the inactive phase of the disease. Because of this variability, several cortisol samples are usually necessary (24-h UFC or nocturnal salivary cortisol) to characterize the cycle, and late-night salivary cortisol is, therefore, the most practical (103).

The low-dose dexamethasone suppression test is not considered the best method, as it can have false negative results when performed in between cycles.

Recently, the utility of capillary (hair) cortisol has been evaluated with the goal of establishing a temporal characterization of the cortisol secretion in cyclic Cushing’s syndrome (104).

***Adrenal incidentaloma***

The screening of Cushing’s syndrome is formally indicated in the subgroup of patients with adrenal incidentaloma because 5.3-9.2% of the patients present subclinical Cushing’s syndrome (105,106). The recommended initial examination is a low-dose dexamethasone suppression test, using a cutoff of 1.8 µg/dL to optimize the sensitivity of the method (27). Other values, such as 5 µg/dL (107,108) or 3 µg/dL in an overnight 3 mg test, increase the specificity of the method but reduce its sensitivity (109).

As an alternative, late-night salivary cortisol can be used, but it has shown less sensitivity when compared to the low-dose dexamethasone suppression test in this subgroup of patients (57,110-113). Finally, with regard to first-line methods, 24-h UFC is the method with the highest number of false negative results, as it is diagnostic only when the disease is overt. Other test results may be useful, such as measurements of ACTH and DHEAS, which may be suppressed in these cases, as well as an attenuated response of ACTH and cortisol in the CRH test (27).

**Figure 1:**
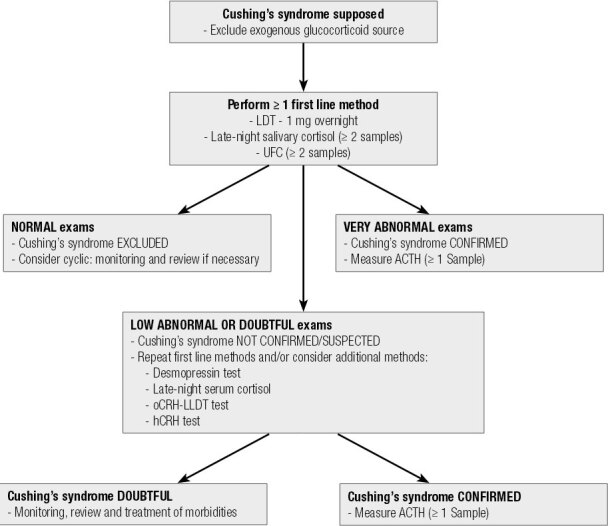
Flowchart of Cushing’s syndrome diagnosis.

LDT: low-dose dexamethasone suppression test with 1 mg overnight; UFC: urinary free cortisol; oCRH: ovine; hCRH: human CRH; LLDT: longer low-dose dexamethasone suppression test (2 mg/day for 48 h).

Figure 1 shows a flowchart for the diagnosis of Cushing’s syndrome.

## DIFFERENTIAL DIAGNOSIS OF ACTH-DEPENDENT CUSHING’S SYNDROME

After a laboratorial confirmation of endogenous Cushing’s syndrome, the subsequent diagnostic approach is to classify the syndrome according to the plasma ACTH levels: ACTH-dependent (ACTH higher than 20 pg/mL: CD *vs*. EAS) or ACTH-independent (ACTH less than 10 pg/mL: adenomas, carcinomas, or adrenal hyperplasia) Cushing’s syndrome. Due to the non-regular secretion of ACTH, it is recommended to perform at least two measurements on different days to confirm the condition (49,114). ACTH values in the range of 10-20 pg/mL are considered indeterminate, and new samples must be ordered.

Due to the scope of this recommendation manuscript, we will only address the differential diagnosis between CD and EAS.

CD represents 86-93% of the cases of ACTH-dependent Cushing’s syndrome (115-117). Because of the high pretest probability of a CD diagnosis, 90% in women and 70% in men, diagnostic methods must ideally show an accuracy higher than 80-90%.

Many methods are used for this purpose, although a triad is usually initially chosen: magnetic resonance imaging (MRI) of the pituitary, a CRH test, and a high-dose dexamethasone suppression test (HDDST). Whenever these three methods are not conclusive, bilateral and simultaneous inferior petrosal sinus sampling (BIPSS) remains the gold standard procedure for the differential diagnosis of CD and EAS (49,114,118-123).

### Pituitary MRI

Pituitary MRI is usually the first exam to be ordered and remains the most important in defining the need for BIPSS. Due to the diagnostic difficulties and the necessity of sensitive methods, computerized tomography of the pituitary is not currently indicated, and a full cranium/brain MRI is considered not ideal for providing slices that are thin enough and focused to the sellar region.

CD is caused by pituitary microadenomas (less than 10 mm) in 80-90% of such cases (5-7).

Conventional MRI spin echoes can present pitfalls such as artifacts (*e.g*., hyposignals of the pituitary parenchyma adjacent to the bony septum insertion of the sphenoid sinus in the sella floor) and the possibility of contrast uptake in the pituitary tumor. Therefore, the sensitivity of the conventional MRI spin echo is 50-60% (124), even for procedures using dynamic MRI.

In cases in which an expansive pituitary macroadenoma (greater than 10 mm) is found, the diagnosis of CD is virtually confirmed. This presumed confirmation is important because this subset of patients may have a poorer response to a CRH test (125) and less suppression in the HDDST (5,7,125,126).

Currently, a maximum diameter of more than 6 mm is suggestive of CD etiology (22,49,118,121,123), especially for patients who respond to a CRH test and present cortisol suppression in the HDDST.

Other secondary findings from the MRI, although nonspecific, may also be helpful in diagnosing a microadenoma, such as deviations from the pituitary stalk, commonly to the opposite side of an expansive lesion, bulging of sella turcica or upper contours of the pituitary parenchyma, hyperintensity in the T2-weighted sequence by small intra-tumor cystic degeneration, and adjacent invasion of a cavernous sinus.

Thus, a patient with concordant tests suggestive of CD, a pituitary image slightly smaller than 6 mm that is also coupled with the above-mentioned suggestive MRI findings, is usually sufficient to establish the diagnosis of a central source of ACTH. Finally, for those who have lesions smaller than 10 mm with or without secondary findings on the MRI but who show negative or inconclusive results on dynamic testing of ACTH and cortisol, BIPSS is recommended to establish or negate the diagnosis of CD.

To increase the pituitary MRI accuracy, other techniques have been sought for diagnostic improvement: spoiled gradient recalled acquisition (SPGR), which increases sensitivity through thinner slices (1 mm) and provides a more focused image resulting in a better soft tissue definition (124,127-129), has been tested, although it also increases the amount of artifacts and false positives. Additionally, 3-Tesla MRI (130-132) and other techniques have also been studied. More studies and a greater availability of these methods are needed to confirm their contribution to the diagnosis of CD.

### CRH test

A CRH test is the best non-invasive dynamic test to differentiate CD from EAS. First identified in the 1980s (133), it has been extensively studied in this context. Most cases of CD respond significantly to CRH (86-93%) (1), whereas EAS patients respond in 5.5-8.2% of cases (115,134). The enhanced responses to CRH in CD are as much due to deranged feedback as they are to an over-expression of CRH receptors.

The test is performed using ovine CRH (oCRH) or human CRH (hCRH). OCRH is the most studied peptide, as it has a more powerful and prolonged stimulus. Most commonly, a positive response is defined as an increase compared to baseline values (peak minus baseline) that is higher than 20% for cortisol and higher than 35% for ACTH with oCRH (135). For evaluations using hCRH, a positive response is considered for increases greater than 14% for cortisol and greater than 105% for ACTH (85,86).

The study that defined the cutoff values for the hCRH test (higher than 14% for cortisol and higher than 105% for ACTH) found a 70% and 85% sensitivity for ACTH and cortisol, respectively, and 100% specificity for each hormone (85).

The test is performed with an IV infusion of 1 µg/kg or 100 µg of ovine or human CRH without prior dexamethasone suppression. The rationale for the test is based on the overexpression of the CRH receptor subtype 1 (CRHR1) in corticotropic tumors when compared to both normal pituitary tissue (136,137) and tumors causing EAS (138). As mentioned previously, due to the high cost and unavailability of CRH, the routine use of this method is limited.

### High-dose dexamethasone suppression test

Of the initial triad, the HDDST is advantageous due to its availability and cost. However, it is the most questioned method in the literature due to its limited accuracy in differentiating between CD and EAS (49,139).

The rationale behind this method is the preservation of negative feedback at higher doses of glucocorticosteroids in patients with corticotropic tumors. However, 25-30% of patients with EAS can also show the same pattern of cortisol suppression, leading to false positive results (116,140).

The HDDST is an old method (> 50 years) and was initially designed to measure cortisol metabolites in 24-h urine (17-OHCS) (141). Currently, it is performed through the measurement of serum cortisol between 8:00 am and 9:00 am before and after a high-dose (8 mg) oral night intake of dexamethasone and is considered suggestive of CD when a reduction of more than 50% is observed compared to the baseline value.

In brief, it can be performed with two protocols: 2 mg of dexamethasone every 6 hours for two days (8 doses – the classic method) or a simplified method with intake of a single 8 mg overnight dose. The sensitivity and specificity of this method varies greatly due to the different protocols of dexamethasone administration used and to the different modes of cortisol analysis (urinary or serum).

To increase the specificity of the method, a more stringent criterion has been proposed for CD diagnosis, namely a greater than 80% cortisol suppression (140,142). Using this criterion and the single intake of 8 mg overnight, one study showed 100% specificity in a small group (*n *= 7) of patients with EAS, in which 28.6% of the patients suppressed cortisol at a level higher than 50%. However, in this same study, only 56% of the 39 patients with CD showed greater than 80% suppression, for a total accuracy of only 63% (140).

Finally, there has been an attempt to combine CRH response and the HDDST to increase the specificity of CD diagnoses. However, even the combination of the methods and the use of the most appropriate cutoff values has not provided enough accuracy to preclude the need for BIPSS in several cases.

### Bilateral and simultaneous inferior petrosal sinus sampling

This method remains the gold standard for the differential diagnosis of ACTH-dependent Cushing’s syndrome with an accuracy of approximately 90-98% (143-145).

**Figure 2:**
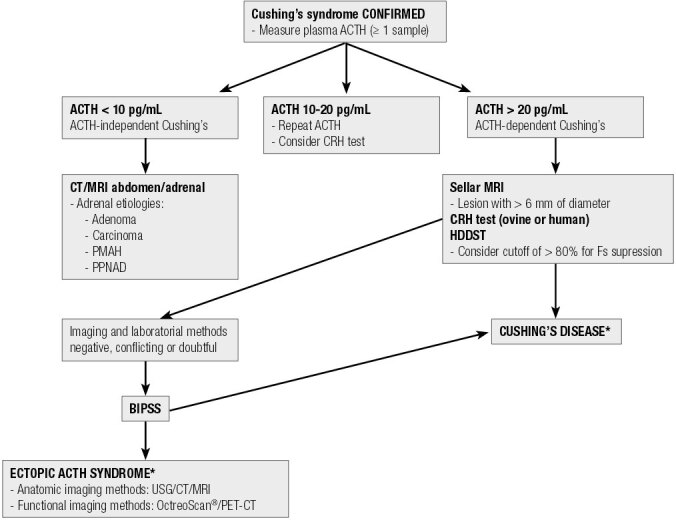
Flowchart for differential diagnosis of ACTH-dependent Cushing’s syndrome

HDDST: high-dose dexamethasone suppression test (8 mg overnight); CT: computed tomography; MRI: magnetic resonance imaging; PMAH: primary macronodular adrenal hyperplasia; PPNAD: primary pigmented nodular adenocortical disease; BIPSS: bilateral and simultaneous petrosal sinus sampling; USG: ultrasound; PET-CT: positron emission tomography-computed tomography; * Even before the definition of Cushing’s disease or EAS, anatomical images of the neck/chest/abdomen/pelvis are commonly obtained to contribute to the identification of the ACTH-producing source.

Bilateral and simultaneous inferior petrosal sinus sampling is indicated in cases where the triad of initial tests is inconclusive or discordant (49,118-123) (Figure 2).

False negatives can occur in approximately 5-10% of cases due to technical difficulties, anatomical variations such as plexiform presentation, unresponsiveness to secretagogues or use of drugs that modulate ACTH secretion. One study reported that false negatives only occurred in cases where the ACTH peak was lower than 400 pg/mL (145).

Fortunately, false positives are rare and can occur in cases of EAS during periods of normocortisolism (cyclical Cushing’s syndrome or use of medical therapy for Cushing’s) or in the rare case of ectopic CRH secretion (146).

This procedure should therefore be carried out in the presence of endogenous active hypercortisolism. Consequently, it is necessary to collect 24-h UFC and/or nocturnal salivary cortisol in the evening of the test or in the preceding days to validate the procedure.

As it is an invasive procedure with potential side effects, the procedure should be performed in referral centers with highly skilled professionals.

Fortunately, the rate of serious complications such as cerebral vascular injury, deep venous thrombosis, pulmonary embolism, subarachnoid hemorrhage or cranial nerve paralysis has been very low or absent in many studies (147-150). The most common complications include bruising at the venipuncture site in 3-4% of the cases (147). Usually, a 5000 IU IV heparin infusion is recommended after the start of venous puncture (151).

The procedure is performed under stimulation of oCRH (145,152), hCRH or desmopressin (144,153,154) at the same doses as those used for dynamic secretion tests. Samples are taken at 0, 3, 5 and 10 minutes after the stimulus, and the peak is usually 3 to 5 min after stimulus. The central to peripheral ACTH ratio, or the “central gradient”, that is suggestive of CD etiology is defined as a ratio greater than 2 at baseline levels and/or higher than 3 at the peak.

Lateralization is defined as an interpetrosal gradient higher than 1.4 (152). However, lateralization is surgically confirmed in only approximately 60-80% of cases (1,155-157).

Furthermore, it is important to note that due to a high pretest probability of CD diagnosis, it is recommended to consider the possibility of a false negative result in cases without a central ACTH gradient.

Several aspects have to be observed during the procedure to ensure a reliable collection, including the following: successful catheterization, as confirmed by visualization of the intercavernous sinuses and contralateral petrosal sinus after contrast infusion; observation of anomalies or asymmetries in the petrosal sinus drainage (123,146,158); and proper processing of the samples collected by storage in previously chilled plastic tubes with EDTA and immediate placement in an ice bath after collection.

Finally, recent studies have verified the prolactin values in BIPSS that can be used to correct for possible false negative gradients (51,123,159,160). The values are initially obtained by calculating the central to peripheral prolactin gradient ipsilateral to the largest gradient of ACTH (baseline), which should be higher than 1.8. When the prolactin values are less than 1.8, an unreliable collection of the inferior petrosal sinus should be considered. Subsequently, a ratio of the ACTH and ipsilateral prolactin gradients with cutoff values higher than 0.8 in one study (51) and higher than 1.3 in another (123) have been suggested as indicative of a CD diagnosis.

Additionally, a recent study showed the utility of prolactin in improving tumor location by using the ratio of the interpetrosal gradient of ACTH and the interpetrosal gradient of prolactin (161). However, unlike the evaluation of prolactin in BIPSS, the role of prolactin evaluation in improving tumor location is still in its infancy, and further studies are thus needed to support its use.

### Desmopressin test

The desmopressin test has been used for the differential diagnosis of ACTH-dependent Cushing’s syndrome since 1993 (87). Subsequently, several studies have shown a response in the majority of patients with CD (~80%) (1). However, patients with EAS eventually also show a response, varying from 27-38% (1,116).

Desmopressin acts on both the AVPR1B (V3 or V1b) (137,162,163) and the AVPR2 (V2) (162,164) vasopressin receptors, which have been documented to be overexpressed in corticotropic tumors when compared to both normal pituitary tissue and tumors causing EAS (138,163,165,166).

The test is performed in the same way as that used to differentiate between Cushing’s syndrome and pseudo-Cushing’s (10 µg IV). An increase in cortisol higher than 20% and in ACTH higher than 35% compared to baseline for both measures is considered predictive of CD, similar to the oCRH test in the differential diagnosis between CD and EAS (135).

However, due to the frequently observed response in patients with EAS, the desmopressin test should not be routinely performed to differentiate between the diagnoses of CD and EAS and should therefore be reserved to distinguish between Cushing’s syndrome and pseudo-Cushing’s or as a secretagogue in the BIPSS procedure.

### Other tests

Other laboratory findings may be helpful in establishing a diagnosis of CD or EAS, although not conclusively: hypokalemia, present in 70% of EAS *vs*. 10% of CD patients due to cortisol mineralocorticoid activity in conditions of enzyme 11β-HSD2 saturation; extremely high plasma ACTH concentrations (> 400 to 500 pg/mL; > 88 to 110 pmol/L) in EAS; positive tumor markers in EAS (examples: calcitonin, gastrin, chromogranin, βhCG, alpha-fetoprotein, CEA, CA 19-9, CA 125) (1,167); and measurement of pro-opiomelanocortin (POMC) and/or ACTH precursors (168,169), which are commonly present in patients with EAS despite the poor availability of these measures.

### Diagnosis of EAS and search for ACTH-producing source

The diagnosis of EAS can be made by identifying the ACTH-producing source through surgical documentation of the lesion with a positive immunohistochemistry for ACTH, clinical and laboratory remission of Cushing’s syndrome after excision of the suspected lesion, or the absence of a center to peripheral ACTH gradient in a reliable BIPSS (not suggestive of a false negative result).

An ectopic source of ACTH can be first recognized by imaging studies (“overt”) or recognized in follow-up with repeated imaging methods (“covert”), although it may also remain occult (8-27%) (115,116,134,170,171).

The most common causes of EAS are intrathoracic (83%) (170), and bronchial/pulmonary carcinoid tumors are currently the most common etiologies (115,116,134,171).

Thus, despite the stepwise diagnostic approach suggested in this manuscript, thoracic and abdominal imaging (CT or MRI) are commonly performed, as an evident suspicious lesion may prevent the need for a BIPSS procedure in a patient without visible pituitary imaging in MRI.

Finally, the search for a peripheral ACTH-producing source by imaging exams is indicated after a negative BIPSS for central gradient. The most common imaging methods are ultrasonography (USG), CT and MRI. These should be requested for the thoracic region (CT or MRI), abdomen/pelvis (CT or MRI), and cervical region (USG).

Somatostatin receptor scintigraphy with Indium (^111^In-DTPA-octreotide, OctreoScan^®^) is an important functional complementary method (172), although its sensitivity is not higher than that of plain images (170,173,174), mainly due to the typically small size of bronchial carcinoid tumors (175).

PET-FDG (^18^F 2-deoxy-D-glucose), a test often used in oncology, may eventually be requested to localize ACTH-secreting tumors. Although some case reports have shown its usefulness (176,177), larger detailed studies have shown that it has no advantage over anatomical tests (170,178), probably due to the low metabolic activity of carcinoid tumors.

New forms of PET such as ^18^F-DOPA-PET, scintigraphy with somatostatin analogues with new radionuclides such as Gallium (^68^Ga) (179,180), and improvements in imaging techniques *per se* may increase the accuracy of functional imaging exams.

Regarding the follow-up of patients with occult EAS with tumors that have not been localized, after proper treatment for hypercortisolism, they should be submitted at least once a year to new anatomical imaging of the cervical and thoracic/abdominal/pelvic regions, with special consideration to the chest, as a ACTH-producing source may appear many years after the onset of symptoms, with bronchial carcinoid being the most common cause (181).

Table 6 summarizes the methods for establishing a differential diagnosis of ACTH-dependent Cushing’s syndrome, and Figure 2 shows a flowchart of this differential diagnosis approach.

**Table 6:** Methods for the differential diagnosis of ACTH-dependent Cushing’s syndrome

Method	Reference value	Sensitivity %	Specificity %	Accuracy %
Sellar MRI (spin echo)	-	50-60	-	-
Ovine CRH test (% increase) (1,115,134)	ACTH > 35% and Fs > 20%	86-93	92-94	-
Human CRH test (% increase) (85)	ACTH > 105% and Fs > 14%	70 and 85	100	-
High-dose dexamethasone suppression test (8 mg overnight) (1)	> 50%	65-100	65-100	-
High-dose dexamethasone suppression test (8 mg overnight) (140)	> 80%	56	100	63
Bilateral and simultaneous petrosal sinus sampling (central to periphery ACTH gradient) (143,144)	Baseline ≥ 2 and/or peak ≥ 3	90-95	~100	90-94

**Other methods**	**Comments**			

Desmopressin test (increase of ACTH > 35% and Fs > 20%)	Low accuracy; should not be used routinely			
K (hypokalemia)	70% EAS vs. 10% Cushing’s disease			
Plasma ACTH	Very high concentrations of ACTH (> 400-500 pg/mL) are suggestive of EAS			
Tumor markers (calcitonin, gastrin, chromogranin, βhCG, alfa-fetoprotein, CEA, CA 19-9, CA125)	They can be measured (serum) or expressed (tissue) in up to 70% of EAS cases			
POMC and/or ACTH precursors	Not available; does not guide the etiologic diagnosis of EAS			

Fs: serum cortisol; EAS: ectopic ACTH syndrome; POMC: pro-opiomelanocortin.
